# Ascorbic Acid 2-Phosphate-Releasing Supercritical Carbon Dioxide-Foamed Poly(L-Lactide-Co-epsilon-Caprolactone) Scaffolds Support Urothelial Cell Growth and Enhance Human Adipose-Derived Stromal Cell Proliferation and Collagen Production

**DOI:** 10.1155/2023/6404468

**Published:** 2023-03-04

**Authors:** Alma Kurki, Kaarlo Paakinaho, Markus Hannula, Jari Hyttinen, Susanna Miettinen, Reetta Sartoneva

**Affiliations:** ^1^Faculty of Medicine and Health Technology (MET), Tampere University, Tampere, Finland; ^2^Research, Development and Innovation Centre, Tampere University Hospital, Tampere, Finland; ^3^Department of Obstetrics and Gynaecology, The Hospital District of South Ostrobothnia, Seinäjoki, Finland

## Abstract

Tissue engineering can provide a novel approach for the reconstruction of large urethral defects, which currently lacks optimal repair methods. Cell-seeded scaffolds aim to prevent urethral stricture and scarring, as effective urothelium and stromal tissue regeneration is important in urethral repair. In this study, the aim was to evaluate the effect of the novel porous ascorbic acid 2-phosphate (A2P)-releasing supercritical carbon dioxide-foamed poly(L-lactide-co-*ε*-caprolactone) (PLCL) scaffolds (scPLCL_A2P_) on the viability, proliferation, phenotype maintenance, and collagen production of human urothelial cell (hUC) and human adipose-derived stromal cell (hASC) mono- and cocultures. The scPLCL_A2P_ scaffold supported hUC growth and phenotype both in monoculture and in coculture. In monocultures, the proliferation and collagen production of hASCs were significantly increased on the scPLCL_A2P_ compared to scPLCL scaffolds without A2P, on which the hASCs formed nonproliferating cell clusters. Our findings suggest the A2P-releasing scPLCL_A2P_ to be a promising material for urethral tissue engineering.

## 1. Introduction

The reconstructive surgery of urethral defects caused by urethral strictures, infections, traumas, or congenital malformations remains a great challenge [1–3]. Small defects are reconstructed with autologous genital flaps. However, larger defects require nongenital tissue grafts or allogenic grafts, in which the reconstruction is highly susceptible to complications [3–5]. Therefore, tissue engineering aims to develop new alternatives for enhanced urethral defect reconstruction. The graft material for a urethral application must be suturable, elastic, and flexible while promoting the regeneration of the urothelium barrier and underlying smooth muscle layer and stromal tissue [6, 7].

Vast research has been conducted to discover a scaffold with adequate mechanical and bioactive properties, yet critical difficulties still remain [7, 8]. Previously, decellularized natural matrices, such as bladder acellular matrix or small intestinal submucosa, have been studied for urethral defect reconstruction [5]. Their advantage is the existence of the natural extracellular matrix (ECM) and good biocompatibility. However, the natural decellularized matrices are nontailorable, have high variability with a lack of large-scale manufacturing, and are prone to immunogenicity regardless of the decellularization process [5, 7]. Therefore, there is an emerging interest in utilizing synthetic biodegradable polymers, such as poly-lactic acid (PLA), poly-glycolic acid (PGA), and polycaprolactone (PCL) for urethral applications [7, 9].

The poly(l-L-lactide-co-*ε*-caprolactone) (PLCL), a copolymer of PLA and PCL, has been previously studied by us and others in several soft tissue engineering applications, such as vascular and urogynaecological research [10–16]. In the PLCL copolymer, the undesired stiffness of PLA [17] and the very slow degradation time of PCL [18] can be tailored to the application's requirements. Since the urethra is a highly elastic tissue subjected to repetitive stretching and contracting forces, the repair material needs to enable its high flexibility and simultaneously support the urethral tubular structure for the time of the tissue regeneration [19, 20]. Therefore, as an elastic and flexible polymer, PLCL is a potential material for urethral applications [17, 21].

In addition to the biomaterial selection, the scaffold design, interconnected porosity, and pore size are highly important for the regenerating tissue. Interconnected pores serve as a route for nutrient transport and allow the migration of cells and tissue in-growth into the scaffold. Moreover, the scaffold fabrication method should enable large-scale manufacturing without leaving any toxic residues. Supercritical carbon dioxide (scCO_2_)-foaming is a rapid, environmentally friendly, and cost-effective method for producing scaffolds with controlled porosity and pore size. Porosity and pore size are controlled by temperature and pressure conditions without requiring any toxic solvents or other nondesired components [22–25].

Previous preclinical studies have demonstrated that cell seeding is beneficial in urethral tissue engineering [19, 26–30]. Most importantly, the use of adipose-derived stromal/stem cells (ASCs) or smooth muscle cells has been shown to enhance angiogenesis and the formation of the urethral stromal tissue during the tissue regeneration, inhibiting the formation of urethral fibrosis [26–29]. Besides ASCs, several cell types have been utilized in urethral tissue engineering research, including fibroblasts [31, 32], smooth muscle cells [27, 33, 34], stromal/stem cells [35–38] along with urothelial cells (UCs) [10, 39–42].

As a native cell type, UCs are frequently studied for urethral tissue regeneration. It is possible to obtain UCs by performing bladder washing without the need for an invasive biopsy [28, 43, 44]. Still, the regeneration of the urethral stroma is highly important for the tissue-engineered urethra. Human ASCs (hASCs) are easily isolated in abundance and are demonstrated to differentiate towards smooth muscle cells [45–47]. As such, they are appealing cell alternatives to smooth muscle cells for forming urethral stromal tissue. Moreover, ASCs and other mesenchymal stem and progenitor cells are capable of reducing inflammation and promoting angiogenesis and intrinsic tissue regeneration in situ [29, 48, 49].

Cell growth and differentiation can be aided by using bioactive molecules. Ascorbic acid (AA) is an essential nutrient and antioxidant that cannot be synthesized by human cells. It plays a central role in multiple cellular processes, such as collagen synthesis where it is a critical cofactor required to form stable collagen fibrils [50, 51]. Supplemented and scaffold-embedded AA has been demonstrated to increase collagen production, cell proliferation, and maturation of various cell types, such as fibroblasts, osteoblast-like cells, and hASCs [52–56]. Particularly, AA and its derivatives enhance the proliferation and differentiation of stem cells into several cell types, such as adipocytes, cardiac myocytes, and osteoblasts [53, 57–59]. However, AA is very unstable in aquatic conditions, so a more stable AA derivative ascorbic acid 2-phosphate (A2P) is commonly used instead [56, 60].

In this study, the aim was to reveal the potential of A2P-embedded scCO_2_-foamed PLCL scaffold (scPLCL_A2P_) for urethral tissue engineering. We studied the effects of scPLCL scaffolds with and without A2P in hUC and hASC monocultures and coculture. Human UCs were cultured to generate differentiated urothelium, and hASCs were cultured to initiate the formation of the urethral stroma. We hypothesized that, while supporting the growth of hUCs, the A2P embedded in scPLCL_A2P_ further enhances the proliferation and collagen production of hASCs and promotes the formation of the urethral stromal tissue compared to a plain scPLCL scaffold. To our knowledge, this is the first study where either scCO_2_-foamed or A2P-embedded scaffolds were studied for urethral applications.

## 2. Materials and Methods

### 2.1. Urothelial Cell Isolation and Culture

The hUCs were isolated from urothelium tissue pieces of three child donors, aged 3 months to 2 years 9 months during elective surgery in the Tampere University Hospital with the approval of the Ethics Committee of Pirkanmaa Hospital District, Tampere Finland (R07160) and written consent from the parents. The isolation protocol for hUCs has been presented in our previous publication [10]. Briefly, the urothelial tissue was cut into small pieces and incubated in a stripping solution of 1% HEPES (Sigma-Aldrich, St. Louis, MO, USA), 0.001% aprotinin (1 kIU/*µ*l; Sigma-Aldrich), 0.1% EDTA (Sigma-Aldrich), and antibiotics (100 U/ml penicillin and 0.1 mg/ml streptomycin, P/S; Lonza, Bio Whittaker, Verviers, Belgium) in HBSS without Ca^2+^ and Mg^2+^ (Invitrogen, Thermo Fisher Scientific, Waltham, MA, USA) overnight at +4°C to loosen the urothelial layer. The urothelium was separated from the stroma and incubated at +37°C water bath for 30 min in 0.1% trypsin solution. The isolated hUCs were suspended in EpiLife culture medium (Gibco by Life Sciences, Thermo Fisher Scientific), supplemented with EpiLife Defined Growth Supplement (EDGS; Gibco by Life Sciences), 0.1% CaCl_2_ (Gibco by Life Sciences) and 0.35% P/S, and cultured on CellBIND T75 flasks (Corning, Sigma-Aldrich) at 37°C in a humified atmosphere of 5% CO_2_. The hUCs used in monoculture experiments were passage 4 (P4) whereas hUCs in coculture experiments were P5.

### 2.2. Adipose-Derived Stromal Cell Isolation and Culture

The hASC were obtained during routine elective surgery in the Tampere University Hospital from three donors, aged between 43 and 71 years, with the approval of the ethics committee of Pirkanmaa Hospital District, Tampere Finland (R15161) and written consent from the donors. The hASCs were isolated as described previously [61, 62]. Briefly, adipose tissue was cut into small pieces and incubated on a shaker in a 37°C water bath for 1 h in 1.5 mg/ml collagenase type I solution. Digested cells were centrifuged and suspended in basic medium (BM) composed of 5% sterile filtered human serum (Biowest, Nuaillé, France) and 1% P/S (Lonza) in Minimum Essential Medium Eagle-Alpha Modification (*α*MEM; Gibco by Life Technologies). The cell suspension was filtered and cultured in Nunc T175 flasks (Nunc, Thermo Fischer Scientific) at 37°C in a humified atmosphere of 5% CO_2_. The expression of surface proteins can be found in Table 1. The expression of surface proteins was assessed after the isolation process in passage 1 (*P*1) to ensure the quality of the isolation protocol and confirming that the isolated cells can be stated as ASCs [63, 64]. The hASCs in monoculture experiments were *P*4 and in coculture *P*5.

### 2.3. Scaffold Manufacturing

The scaffold manufacturing process was similar to a previously published protocol [65]. Briefly, the porous scPLCL_A2P_ scaffolds were manufactured by melt-mixing 8 wt-% of A2P (Sigma-Aldrich Chemie Gmbh, Steinheim, Germany) to PLCL 70L/30CL (PLCL 7015, Corbion Purac BV, Gorinchem, The Netherlands) in a twin-screw extrusion process, after which the material was foamed by scCO_2_ (Waters Operating Corporation, Milford, MA, USA) using high pressure and temperature of 90°C. Porous scPLCL scaffolds were manufactured similarly but without the melt mixing of A2P. To acquire the scaffolds, the foamed rods were cut into 8 mm diameter discs with a thickness of 3-3.5 mm. The scaffolds were gamma-irradiated prior to cell culture.

### 2.4. Scaffold's Porosity and Assessment with X-ray Microcomputed Tomography Imaging

The scPLCL and scPLCL_A2P_ scaffolds (*n* = 3) were analysed with X-ray microtomography. MicroXCT-400 (Carl Zeiss X-ray Microscopy, Inc., Pleasanton, CA, USA) device was used with the X-ray tube voltage of 60 kV and a current of 167 *µ*A. 1601 projections were taken with 1 sec exposure time. The pixel size was 5.64 *µ*m. Acquired projection data were reconstructed with Zeiss' XMReconstructor software. For the pore analysis, a 3.8 × 3.8 × 1.2 mm volume was selected from each scaffold. The image processing and the visualizations were made with the Avizo 3D 2021.2 software (Thermo Fisher Scientific, Waltham, MA, USA). The material of the scaffold visible in the images and denser A2P particles were segmented with manual thresholding. The interconnectivity of the scaffold pores was evaluated with a Matlab program that uses the pore size data calculated with the BoneJ plugin [66] providing size distribution of the interconnected pores. The analysis procedure is described in more detail in [67]. For accuracy, a minimum particle size of 11.28 *µ*m (twice the used pixel size of 5.64 *µ*m) was used for interconnectivity and A2P-particle size calculations.

### 2.5. Cell Seeding on Scaffolds

**Figure 1:**
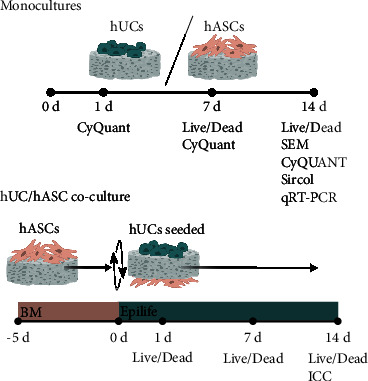
Research timeline for hUC and hASC monocultures and hUC/hASC coculture. The hUC and hASC monocultures were treated similarly. In coculture, the hASCs were seeded in basic medium (BM) five days prior to seeding the hUCs on the opposite side of the scaffold and changing the coculture medium to EpiLife. The time points for mono- and cocultures were d1, d7, and d14.

Before cell seeding, the scaffolds were prewetted in culture medium, EpiLife for hUCs, and BM for hASCs, for 24°h at +37°C. The prewetted scaffolds were placed into 24-well plates for the cell experiments. For the monoculture study, 150 000 hUCs or hASCs were seeded on the surface of the scaffold in a plating volume of 50 *µ*l. The cells were left to adhere in +37°C incubator for 2 h before adding 1 ml medium to each well. For the coculture experiment, 150 000 hASCs in 50 *µ*l of medium were seeded on the other side of the scaffold and precultured for five days in BM to allow cell expansion. Thereafter, 150 000 hUCs in 50 *µ*l of medium were seeded on the opposite side of the scaffold, and the medium was switched to EpiLife medium for the coculture. A visualization of the research timeline is presented in Figure 1. The cell-seeded scaffolds were cultured in a humidified +37°C incubator, and the medium was changed three times a week until analysis. The analyses were performed at d1, d7, and d14 time points.

### 2.6. Cell Viability and Proliferation

Cell viability in monocultures and coculture was determined at d1, d7, and d14 time points with qualitative live/dead fluorescent staining (Invitrogen, Life Technologies) as described previously [10]. Briefly, the samples were incubated in 3.75*∗*10^−5^ *µ*M ethidium homodimer-1 (EthD-1) and 0.5 *µ*M calcein acetoxymethyl ester (Calcein-AM) solution for 1 h in RT. Unseeded scaffolds were used as negative controls. Samples were imaged with a fluorescence microscope (Olympus IX51S8F-2; camera DP71), and the images were processed with Adobe Photoshop 2022-software by adjusting contrast and brightness.

On d1, d7, and d14 time points, the relative cell number in hUC and hASC monocultures was assessed with a quantitative CyQUANT Cell Proliferation Assay kit (Invitrogen, Life Technologies). The assay was repeated with three donor lines for each cell type with three parallel samples using technical triplicates in the assay run (*n* = 27). To lyse the cells, 0.1% Triton x-100 (Sigma-Aldrich) in DPBS was added to each sample, and the lysates were stored at −70°C. For analysis, CyQUANT GR dye and lysis buffer were added to the thawed lysates, and fluorescence at 480/520 nm wavelength was measured with a Wallac Victor microplate reader (PerkinElmer Life and Analytical Sciences, Wallac, Turku, Finland). Cell numbers were calculated relative to the scPLCL d1 sample.

### 2.7. Scanning Electron Microscopy

Scanning electron microscopy (SEM) was used to assess the cell attachment and morphology after 1, 7, and 14 d of cell culture in hUC and hASC monocultures using one donor line from each cell type. Samples were fixed with 5% glutaraldehyde (Sigma-Aldrich) in 0.1 M phosphate buffer (pH 7.4; Sigma-Aldrich) for 48 h at RT. The sample drying procedure with hexamethyldisilazane (HMDS; Sigma-Aldrich) was performed as described by Sartoneva et al. [68]. Samples were dehydrated with increasing series of ethanol (Altia Oyj, Helsinki, Finland) concentrations (30, 50, 70, 80, 90, 95, and 100%) after which samples were dried with ascending series of HMDS in 100% ethanol (1 : 2, 2 : 1 and twice 100% HMDS). After HMDS was evaporated, samples were carbon sputtered and imaged with SEM (Zeiss ULTRAplus, Oberkochen, Germany).

### 2.8. Total Collagen Content

Sircol soluble collagen assay (Biocolor, Carrickfergus, UK) was used to quantify the amount of total soluble collagen in hUC and hASC monocultures at d14 time point. The analysis was performed for three donor lines with three parallel samples and two technical replicates for each cell type (*n* = 18). The assay was carried out as described previously [69]. The samples were incubated in ice-cold 0.1 mg/ml pepsin (Sigma-Aldrich) in 0.5 M acetic acid for 4 h at +4°C to extract the acid-soluble collagen. Sample suspensions were moved to separate tubes, and Sircol dye reagent, consisting of picric acid and Sirius red, was added to each sample. After 30 min incubation at RT, the samples were pelleted and resuspended to ice-cold Sircol Acid-Salt Wash Reagent to remove any unbound dye. 0.5 M sodium hydroxide solution was then added to resolubilize dyed collagen. Dye intensity was measured with a Wallac Victor microplate reader at 540 nm wavelength.

### 2.9. Quantitative Real-Time Polymerase Chain Reaction

Quantitative real-time polymerase chain reaction (qRT-PCR) was used to analyse the relative expression of cytokeratin (CK) 7, CK8, CK19, uroplakin (UP) Ia, and UPIb in hUCs and relative expression of collagen type I (COL I), COL III, *α*-smooth muscle actin (*α*SMA), and elastin in hASCs. The assay protocol is described in more detail in [70]. For each donor line, cells from three parallel sample wells were pooled for mRNA isolation. Briefly, total RNA was isolated after 14 d of cell culturing with the NucleSpin RNA II purification kit by following the manufacturer's protocol (Macherey-Nagel GmbH & Co. KG, Düren, Germany). Isolated total RNA was then reverse transcribed to cDNA using High-Capacity cDNA Reverse Transcriptase Kit (Applied Biosystems, Foster City, CA). For the qPCR run, 50 ng of sample cDNA was added to the mixture of Power SYBR Green PCR Master Mix (Thermo Fisher Scientific) and 360 nM forward and reverse primers. Each sample was run in duplicates (*n* = 6). Used primers are listed in Table 2. The qRT-PCR run was performed using the ABI PRISM 7300 sequence detection system (Applied Biosystems). Acquired data were normalized to housekeeping gene human ribosomal protein lateral stalk subunit P0 (hRPLP0), and the relative amount of mRNA was calculated using a previously described mathematical model [71]. The ratio of hASC COL I/III mRNA was determined by using the qRT-PCR cycle threshold (Ct) values for COL I and COL III mRNA.

### 2.10. Cytochemical and Immunofluorescent Staining of Cocultures

Cytochemistry and immunofluorescence staining of acidic and basic cytokeratins detected in epithelial cells (mouse cytokeratin pan type I/II antibody cocktail, MA5-13156, 1 : 250; Thermo Fisher Scientific), UPIII (rabbit anti-UPK3A; orb248591, 1 : 100; Biorbyt, Cambridge, United Kingdom), and F-actin cytoskeleton organization (phalloidin-tetramethylrhodamine B isothiocyanate, P1951, 1 : 500; Sigma-Aldrich) were performed for cocultures at d14 time point (*n* = 2). Samples were fixed with 0.2% Triton x-100 (Sigma-Aldrich) in 4% PFA (Sigma-Aldrich) and incubated overnight at +4°C with primary antibodies. Secondary antibodies and phalloidin were added the next day and incubated for 45 min at +4°C. A mixture of Alexa 488 goat anti-mouse IgG1 (A21121, 1 : 400 green fluorescence; Thermo Fisher Scientific) and phalloidin was used for pancytokeratin and F-actin costaining. Alexa 594 goat anti-rabbit IgG (A11037, 1 : 300, red fluorescence; Thermo Fisher Scientific) was used for UPIII staining. Cell nuclei were stained with DAPI (1 : 2000, blue fluorescence; Sigma-Aldrich). Monocultures of hUCs and hASCs cultured in EpiLife medium on polystyrene well bottom served as control samples. Cell-seeded scaffolds without primary antibodies and nonseeded scaffolds were used to exclude any nonspecific staining of the secondary antibodies or false staining caused by the scaffold material, respectively. Samples were imaged with a fluorescent microscope (Olympus IX51), and images were processed with Adobe Photoshop 2022 software by adjusting contrast and brightness.

### 2.11. Statistical Analysis

Quantitative results were analysed with IBM SPSS Statistics software (Version 26, IBM Corp., Armonk, USA) using statistical tests depending on the number of samples. Mann-Whitney test or Kruskall-Wallis test for nonnormally distributed data was used for Sircol assay results (*n* = 18), CyQUANT (*n* = 18–27) and qRT-PCR results (*n* = 6). Bonferroni post hoc tests were included. Significance level *p* < 0.05 was considered as significant. All quantitative assays were performed for three donor lines for each cell type, and results from technical replicates of each sample were used in the calculations and statistical tests.

## 3. Results

### 3.1. Both scPLCL_A2P_ and scPLCL Scaffolds Present High Porosity with High Interconnectivity

**Figure 2:**
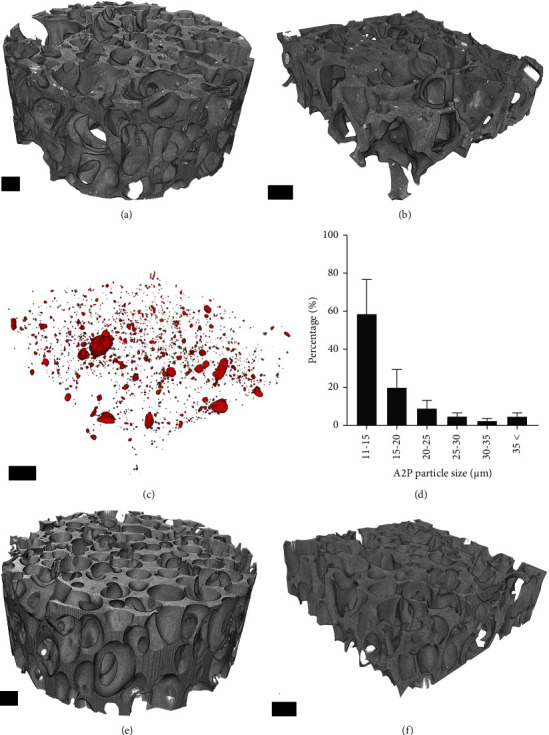
Micro-CT images of the scPLCL_A2P_ and scPLCL scaffolds. Overall structure of scPLCL_A2P_ scaffolds (a), volume (3.8 × 3.8 × 1.2 mm) of scPLCL_A2P_ used in the porosity and A2P particle distribution measurements (b), distribution of the A2P particles in scPLCLas (c), particle size distribution (*n* = 3) (d), overall structure of scPLCL scaffolds (e), and volume (3.8 × 3.8 × 1.2 mm) of scPLCL used in the porosity measurements (f). Scale bar 500 *µ*m.

Micro-CT was used to assess scaffold architecture and pore interconnectivity. In addition, the distribution of A2P particles in the scPLCL_A2P_ was measured. Overall visualization of the scPLCL_A2P_ and scPLCL scaffolds is represented in Figure 2. The image-based assessment revealed that the A2P particles are evenly distributed in scPLCL_A2P_ (Figure 2(c)). Particles larger than 11.28 *µ*m were measured, and approximately 60% of A2P particles had a diameter between 11.28–15 *µ*m.

**Figure 3:**
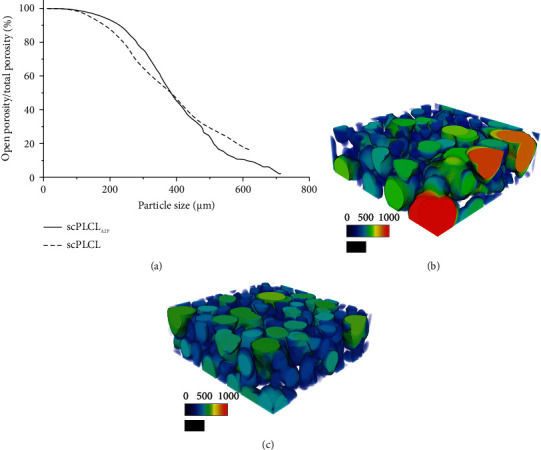
Micro-CT analysis of the scaffold pore sizes and interconnectivity. (a) Scaffold pore interconnectivity as a function of particle size capable of passing through the interconnected pores entering from outside of the scaffold. Distribution of pore sizes in scPLCL_A2P_ (b) and scPLCL (c). Color scale represents the size of a particle capable of entering a pore (0–1000 *µ*m). Scale bar 500 *µ*m.

Average porosity was slightly lower in scPLCL_A2P_ (63%) than in scPLCL (69%), yet the average pore size was higher in scPLCL_A2P_ (490 ± 200 *µ*m) than in scPLCL (400 ± 140 *µ*m) (Table 3). In Figure 3, open porosity (*y*-axis) represents the percentage of interconnected scaffold pores that could be passed through by various sizes of spherical particles (*x*-axis) from outside of the scaffold. For example, particles with diameter ≤205 *µ*m can pass through 90% of the pores in both scPLCL_A2P_ and scPLCL scaffolds (Figure 3(a)). One noticeably differing scPLCL_A2P_ scaffold has been removed from the results.

### 3.2. A2P in scPLCL_A2P_ Supports hUC and hASC Viability

**Figure 4:**
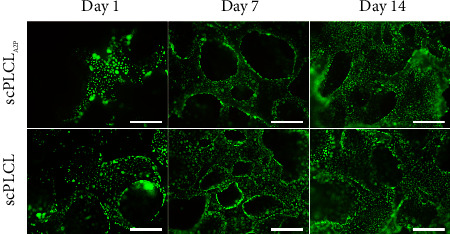
Viability of hUCs monoculture on scPLCL_A2P_ and scPLCL at d1, d7, and d14. Viable cells are shown green and dead cells red. Cell growth seems parallel on both scaffolds. Scale bar 500 *µ*m.

**Figure 5:**
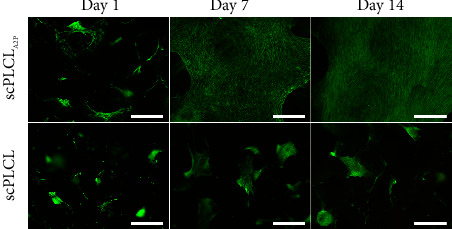
Viability of hASCs monoculture on scPLCL_A2P_ and scPLCL at d1, d7, and d14. Viable cells are shown green and dead cells red. The hASCs appear to be spreading more on scPLCL_A2P_ than on scPLCL. Scale bar 500 *µ*m.

Cell viability was evaluated with Live/Dead staining on d1, d7, and d14 for monocultures and cocultures. Both hUCs and hASCs remained viable during the 14-d assessment period in monocultures. No differences were detected in hUC viability on scPLCL_A2P_ and scPLCL (Figure 4). The hASCs spread rapidly on scPLCL_A2P_ and covered the whole scaffold surface after 7 d, continuing to form dense cultures between d7 and d14 (Figure 5). On scPLCL, the hASCs did not spread widely along the scaffold surface but rather formed viable cell clusters during the 14-d cell culture.

**Figure 6:**
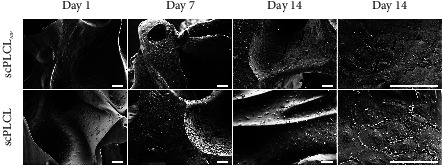
Scanning electron microscope (SEM) images showing the morphology of hUCs in monoculture on scPLCL_A2P_ and scPLCL at d1, d7, and d14. Spreading of hUCs seems similar on both scaffolds, yet on d14, the hUCs on scPLCLas seem to have acquired more structural apical surface (arrows). Scale bar 100 *µ*m.

**Figure 7:**
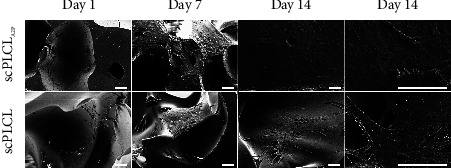
Scanning electron microscope (SEM) images showing the morphology of hASCs in monoculture on scPLCL_A2P_ and scPLCL at d1, d7, and d14. The hASCs on scPLCL_A2P_ appear to spread more along the scaffold compared to scPLCL. Scale bar 100 *µ*m.

Closer inspection of the monocultures with SEM on d1, d7, and d14 shows more detailed differences in the cell attachment and morphology on different scaffolds. The hUCs cultured on both scPLCL_A2P_ and scPLCL presented a typical urothelial morphology with defined cell borders, and the hUCs spread evenly on scaffold surface (Figure 6). On d14, hUCs on scPLCL_A2P_ had more cuboidal morphology with visible cell borders, and some hUCs had acquired apical irregularities. Instead, the hUCs on scPLCL had flattened appearance along the material surface. Uniform and aligned sheet of hASC covered the scPLCL_A2P_ scaffold surface, whereas on scPLCL, the hASCs were clustered together, supporting the findings of the hASC monoculture Live/Dead viability assay (Figure 7).

**Figure 8:**
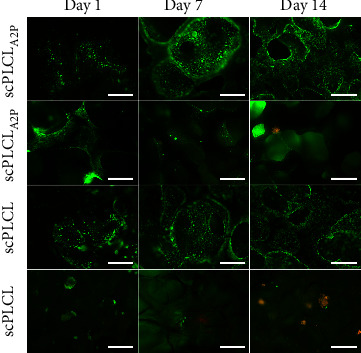
Fluorescent images showing the viability of hUCs and hASCs in hUC/hASC coculture on scPLCL_A2P_ and scPLCL at d1, d7, and d14 time points. Viable cells stain green and dead cells red. Cell growth of hUCs remains similar on both scaffolds. Viability of hASCs is supported better on scPLCL_A2P_, whereas hASCs on scPLCL seem to diminish during the coculture. Scale bar 500 *µ*m.

According to the Live/Dead staining of hUC/hASC coculture, no distinctive difference was detected in the hUC viability and spreading between the scPLCL_A2P_ and scPLCL scaffolds (Figure 8), whereas more hASCs appear to be visible on scPLCL_A2P_ scaffold. After commencing coculture in Epilife medium, hASC growth on scPLCL_A2P_ seems to have suppressed, yet the cells remain viable. Meanwhile, the hASCs on scPLCL formed cell clusters similar to monoculture, and after switching to Epilife medium, there seems to be more dead cells compared to scPLCL_A2P_.

### 3.3. scPLCL_A2P_ Significantly Increased the Proliferation of hASCs

**Figure 9:**
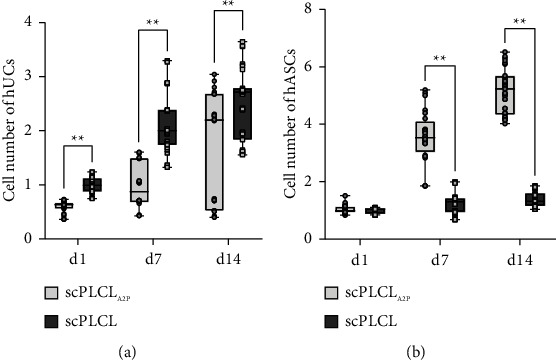
Cell number of hUC and hASC monocultures relative to d 1 scPLCL CyQUANT mean result (*n* = 18–27). Relative hUC number (a) was significantly higher on scPLCL than on scPLCL_A2P_ at each time point (d1–d 7 *p* < 0.001, d1–d14 *p*=0.009). On scPLCL_A2P_, the relative hUC number significantly increased between d1–d7 (*p*=0.022) and d1–d14 (*p*=0.001). On scPLCL, the hUC number increased d1–d7 and d1–d14 (*p* < 0.001), but no statistical significance was detected between d7–d14 (*p*=0.823). Relative number of hASCs (b) was similar on both scaffolds on d 1 (*p*=0.27), but significantly higher on scPLCLas than on scPLCL on d7 and d14 (*p* < 0.001). During the assessment period, relative hASC number on scPLCL_A2P_ increased between each time point (d1–d7 *p* < 0.001, d1–d14 *p* < 0.001, d7–d14 *p* < 0.003). On scPLCL, hASC number increased between d1–d7 (*p*=0.002) and d1–d14 (*p* < 0.001). (^*∗∗*^=*p* < 0.01).

The cell proliferation of hUCs (*n* = 18–27) and hASCs (*n* = 27) on scPLCL_A2P_ and scPLCL was assessed with CyQUANT by measuring the relative amount of DNA in the samples at d1, d7, and d14 time points (Figure 9). The number of hUCs was lower on scPLCL_A2P_ than on scPLCL at each time point (d1 *p* < 0.001, d7 *p* < 0.001, and d14 *p*=0.009). The number of hUCs increased between d1, d7 (*p*=0.022), and d1–d14 (*p*=0.001). On scPLCL, hUC number increased d1–d7 and d1–d14 (*p* < 0.001). No statistical significance in hUC amount between d7–d14 was detected on either scaffold. The number of hASCs was significantly higher (*p* < 0.001) on scPLCL_A2P_ compared to scPLCL at d7 and d14 time points. Furthermore, as a function of time, the hASC proliferation on scPLCL_A2P_ increased significantly from d1 to d7 (*p* < 0.001), from d1 to d14 (*p* < 0.001), and between d7 and d14 (*p*=0.003). The cell number of hASCs on scPLCL increased between d1 and d7 (*p*=0.002) and from d1 to d14 (*p* < 0.001). No significant increase in hASC number on scPLCL was detected between d7 and d14 (*p*=0.27).

### 3.4. Collagen Production by hASCs Significantly Increased on scPLCL_A2P_

**Figure 10:**
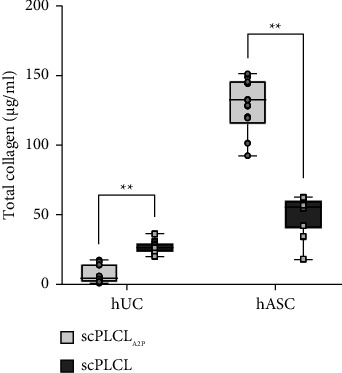
Total amount of collagen present in hUC and hASC monocultures on d 14 (*n* = 18). For hUCs, the amount of total collagen was higher on scPLCL compared to scPLCL_A2P_, whereas for hASCs, the total collagen amount was significantly higher on scPLCL_A2P_. (^*∗∗*^=*p* < 0.01).

The total acid- and pepsin-soluble collagen content of samples was measured with Sircol collagen assay after 14 d of cell culture (Figure 10). Total amount of collagen in hUC monoculture (*n* = 18) was significantly lower (*p* < 0.001) on scPLCL_A2P_ (7.3 ± 6.3 *µ*g/ml) than on scPLCL (26.9 ± 4.5 *µ*g/ml) whereas the hASCs (*n* = 18) cultured on scPLCL_A2P_ produced significantly more collagen (129.4 ± 19.9 *µ*g/ml, *p* < 0.001) compared to scPLCL (49.6 ± 14.2 *µ*g/ml). The hASCs on scPLCL_A2P_ produced approximately 17 times more collagen than hUCs. However, on scPLCL, the difference was only two times higher for hASCs.

### 3.5. scPLCL_A2P_ Supports hUC Phenotype and Enhances hASC *α*SMA and COL III Expression

**Figure 11:**
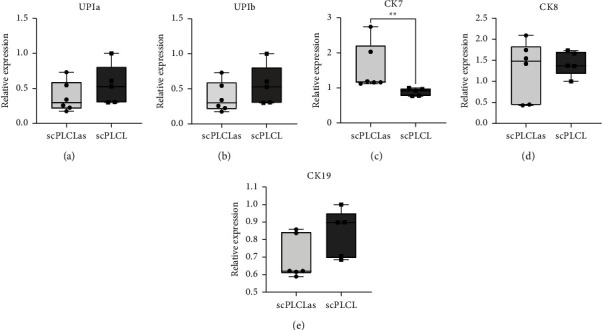
The hUC expression of epithelial markers in monoculture on d14 relative to d1 results of a single used donor hUC line. The expression of CK7 mRNA was significantly increased on scPLCL_A2P_ (c). (*p*=0.004) compared to scPLCL. No significant difference was detected in the expressions of UPIa (a) (*p*=0.329), UPIb (b) (*p*=0.931), CK8 (d) (*p*=1.0), or CK19 (e) (*p*=0.52) between scaffolds. (*n* = 6, ^*∗∗*^=*p* < 0.01).

**Figure 12:**
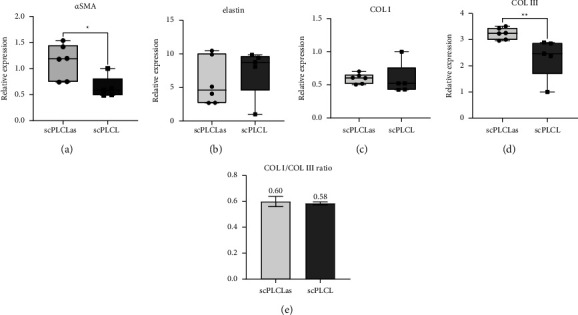
The hASC expression of stromal markers in monoculture on d14 relative to d1 results of a single used donor hASC line. The expression of COL III (d) and *α*SMA (a) mRNA was increased on scPLCL_A2P_ (*p*=0.004 and 0.017, respectively). No significant difference in COL I (c); (*p*=0.429) or elastin (b); (*p*=0.931) mRNA was detected between the scaffolds. Ratio of the expressed COL I/COL III mRNA (e) was similar on both scaffolds. (*n* = 6, ^*∗*^=*p* < 0.05, ^*∗∗*^=*p* < 0.01).

The expression of specific epithelial genes in hUC and stromal genes in hASC monocultures was measured with qRT-PCR on d14 (Figures 11 and 12). The hUC expression of CK7 was significantly increased (*p*=0.004) on scPLCL_A2P_ compared to scPLCL. No other statistical significances were detected. However, relative mRNA amounts of urothelial maturation markers UPIa and UPIb both seem slightly higher on scPLCL. The expression of CK8 and CK19 appears similar on both scaffolds.

The expression of stromal markers *α*SMA, elastin, COL I, COL III, and ratio of expressed COL I/III was determined in hASC monocultures. The amounts of *α*SMA and COL III mRNA were significantly increased (*p*=0.017, *p*=0.004, respectively) in hASCs cultured on scPLCL_A2P_ compared to scPLCL. No statistical significances were detected in elastin or COL I expression, and the mRNA amounts appear parallel on both scaffolds. Average ratios of hASC COL I/III mRNA amounts were similar between the scaffolds, 0.6 on scPLCL_A2_ and 0.58 on scPLCL, when examining the Ct-values of the PCR run. No statistical analysis could be done for the COL I/III ratios, as the sample size (*n* = 3) is too small for reliable statistical analysis.

### 3.6. In Coculture, hUCs Stained for UPIII and hASCs Maintained Myogenic Capabilities on Both Scaffolds

**Figure 13:**
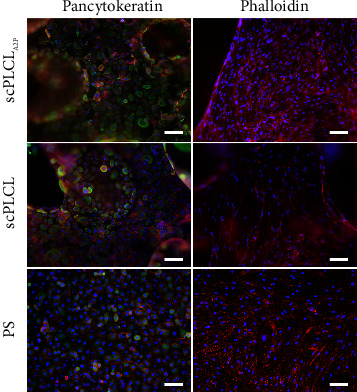
Immunofluorescent staining for pancytokeratin (green) and cytochemical staining for F-actin (red) in hUC/hASC coculture on d 14 on scPLCL_A2P_ and scPLCL. The hUCs on the left panel and the hASCs on the right panel. The PS served as a control material. Scale bar 100 *µ*m.

**Figure 14:**
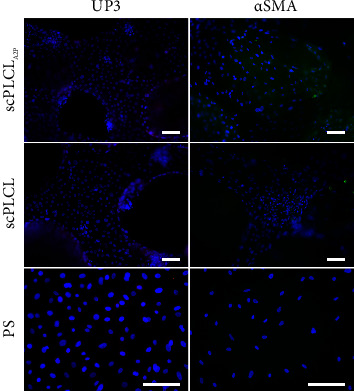
Immunofluorescence staining for UPIII (red) in hUCs and for *α*SMA (green) in hASCs in hUC/hASC coculture on d 14 on scPLCL_A2P_ and scPLCL. hUCs on the left panel and hASCs on the right panel. Control stainings were performed with hUC and hASC monocultures on PS. The hUCs on all materials stain for UPIII. For hASCs, *α*SMA is present on all materials, yet it seems more abundant on scPLCL_A2P_. Scale bar 100 *µ*m.

Cell maturation and phenotype maintenance in hUC/hASC coculture on scPLCL_A2P_ and scPLCL were examined with cytochemical and immunofluorescent staining on d14. The costaining of F-actin and pancytokeratin, the acidic and basic cytokeratins detected in epithelial cells, indicates that the hUCs maintained their epithelial phenotype and had similar cortical actin cytoskeleton organization on both scaffolds (Figure 13). Interestingly, the late hUC maturation marker UPIII was stained positive in hUCs on both biomaterials and faintly on PS although the staining was more of a hue than clearly defined area (Figure 14). The actin cytoskeleton of hASCs was organized and aligned on scPLCL_A2P_ and slightly disorganized on scPLCL. Furthermore, *α*SMA staining in hASCs was superior on scPLCL_A2P_ compared to scPLCL, and very minor staining was detected on control PS.

## 4. Discussion

The surgical repair of urethral defects currently lacks optimal repair methods as the operations are prone to complications, and suitable autologous donor tissue is scarce [3–5]. To overcome such issues, tissue engineering can be utilized to design biomaterials to mimic the mechanical properties of natural tissues, while also providing optimal niche for tissue regeneration. In this study, we compared two supercritical CO_2_-foamed PLCL-based scaffolds, A2P-embedded scPLCL_A2P_, and plain scPLCL, for urethral application. We cultured hUCs and hASCs in mono- and cocultures on porous scPLCL_A2P_ and scPLCL scaffolds to determine the effect of A2P on hUC maturation and on the capability of hASCs to form urethral stromal layer.

Porous scaffolds with interconnected pores allow cell migration into the scaffolds for tissue ingrowth. In this study, we used the process of scCO_2_-foaming to produce porous scPLCL_A2P_ and scPLCL scaffolds. In scCO_2_ foaming, the porosity can be adjusted by altering the time, temperature, and pressure during the fabrication process, and no toxic solvents are required [72, 73]. Porous scaffolds produced with scCO_2_-foaming have previously shown promising results in multiple tissue engineering studies, including in cartilage [22], vaginal [68], and bone [74, 75] applications.

The *µ*CT imaging was performed to show similar scaffold porosity for scPLCL_A2P_ (63%) and scPLCL (69%), with an average pore size of 490 ± 200 *µ*m and 400 ± 140 *µ*m, respectively. The measured porosity was in the same range as in previous publications using similar scCO_2_-foamed PLCL scaffolds [65, 67, 68]. Most importantly, our findings show that the incorporation of A2P did not affect the scaffold porosity, and the pore sizes of both scPLCL_A2P_ and scPLCL are sufficient for cell migration enabling tissue ingrowth. Here, our results indicate that particles up to approximately 400 *µ*m in diameter can enter 50% of the pores in both scPLCL_A2P_ and scPLCL scaffolds, whereas particles up to 200 *µ*m can enter 90% of the pores in both scaffolds. Furthermore, hASCs have been demonstrated to migrate into similar scCO_2_-foamed PLCL scaffolds with a pore size of 350–660 *µ*m [67].

Previously, porous scaffolds embedded with various bioactive components, such as hydroxyapatite and growth factors, have been investigated for tissue engineering applications [75–79]. We wanted to study scPLCL-embedded A2P, as AA has been reported to increase cell proliferation, and it is a critical factor in human collagen synthesis [51, 58]. The scPLCL_A2P_ scaffolds used in this study were incorporated with 8wt-% of A2P. Our previous drug release study using similar porous PLCL scaffolds with 8wt-% A2P revealed that approximately 65% of embedded A2P was released in 37°C buffer solution during the first week. During the first two weeks, the A2P concentration in solution was 6–85 *µ*g/ml which corresponds to 20–260 *µ*M [65]. Similarly, porous PLGA scaffolds embedded with A2P released 52–66% of A2P during the first 9 days [80]. In addition, electrospun PLA scaffolds were shown to release 50% of the incorporated A2P in distilled water within the first 24 h [60]. Previously, 100 *µ*g/ml AA concentration was reported to be most favourable for hASCs whereas concentrations above 300 *µ*g/ml showed cytotoxic effects [81].

To our knowledge, this is the first study to assess the effect of scaffold embedded A2P on hUC growth and phenotype. The hUCs in monoculture remained viable and proliferated on both scaffolds with no dead cells visible. No apparent difference in hUC viability or spreading could be detected in the Live/Dead assay between scPLCL_A2P_ and scPLCL. Whereas visually the differences in the hUC proliferation on scPLCL_A2P_ and scPLCL scaffolds could not be detected, the quantitative CyQUANT proliferation assay revealed significantly higher hUC amount on scPLCL than on scPLCL_A2P_ at every time point.

We utilized SEM to further study the morphology of hUCs cultured on scaffolds. In SEM images, the hUCs had polygonal cell morphology with well-defined cell borders on both scPLCL_A2P_ and scPLCL. Especially on scPLCL_A2P_, the hUCs had acquired distinctive cell boundaries, possible precursors of tight junction rings. In a natural urothelium, well-defined tight junction rings can be detected around mature umbrella cells, the cells on the mucosal surface of a matured urothelium [82]. In addition, we detected that some hUCs on scPLCL_A2P_ had acquired irregular apical surface which suggests a possible presence of microvilli or urethral plaques, both signs of the UC maturation [82–84]. Formation of tight junctions and presence of microvilli have also been previously reported in tissue engineered urothelium [85, 86]. Therefore, even though the hUC proliferation on scPLCL_A2P_ was significantly lower when compared to scPLCL, the morphology of hUCs on scPLCL_A2P_ could suggest further signs of hUC maturation compared to the hUCs on scPLCL scaffolds.

In addition, our results indicate that the hUCs retained their urothelial phenotype on scPLCL_A2P_ and scPLCL. Importantly, the monocultured hUCs on both scaffolds expressed the UC maturation markers UPIa and UPIb, the major components of the urethral plaques [83, 87]. Particularly, the hUCs on both scPLCL_A2P_ and scPLCL scaffolds expressed the cytokeratins CK7, CK8, and CK19, which are present in the cell layers of a mature urothelium [83, 88]. The amount of expressed CK7 mRNA was significantly higher on scPLCL_A2P_ compared to scPLCL. Even though CK7 is present in all layers of the urethral urothelium, it has been reported to have a critical role in mature umbrella cells and therefore could further indicate the maturation of UCs [88, 89]. We have also previously shown that hUCs cultured on PLCL membranes maintain their urothelial phenotype and express the cytokeratins CK7, CK8, and CK19 [10] and that vaginal epithelial cells on a scaffold similar to the scPLCL also expressed UPIa, UPIb, and UPIII [68].

Along with the mature urothelium, the regeneration of stromal tissue and smooth muscle layers is critical for functional urethra [26–29], and therefore, we utilized hASC for the stromal compartment in our study. A2P in scPLCL_A2P_ had a noticeable effect on hASC viability and proliferation. Our results show how cells in the hASC monoculture proliferated and spread densely over the scPLCL_A2P_ scaffold, whereas on scPLCL, the hASCs formed clusters. The dense spreading of hASCs on scPLCL_A2P_ and clustering on scPLCL was also visible in SEM imaging. Such clustering on scPLCL scaffolds was also seen in our previous study using human vaginal stromal cells [68]. One possible explanation for cell clustering could be the hydrophobicity of the PLCL [90] hindering the hASC attachment and forcing them to form clusters with each other. The incorporation of A2P could decrease the hydrophobicity of PLCL and therefore allow better cell attachment, as also demonstrated for PLA scaffolds [60]. The striking effect on hASC proliferation was also observed in CyQUANT proliferation assay, where the hASCs on scPLCL_A2P_ kept proliferating the entire 14-d assessing period, and the number of hASCs remained superior when compared to scPLCL at each time point. Therefore, in contrast to hUCs, the A2P in scPLCL_A2P_ strongly enhances the hASC proliferation.

Even though A2P enhanced the hASC proliferation in our study, too high concentrations of AA or A2P in cell culture have been suggested to lead to cytotoxicity and reduced viability [58, 81, 91], although contradictory reports have been published. For hASCs, cytotoxicity of supplemented A2P appears to be dose-dependent and could be prevented by increasing the cell density. Moreover, AA was more cytotoxic than A2P. Concentration of 250 *µ*M A2P supplemented in the cell culture medium has been reported to be beneficial for hASC proliferation, stemness, and collagen synthesis [53], but cytotoxicity has also been detected with the same 250 *µ*M concentration [91]. The 250 *µ*M concentration has also shown to increase proliferation of bone marrow-derived mesenchymal stem cells [58].

Collagen is the major component of stromal ECM, and both AA and A2P can stimulate its production and enhance the collagen mRNA expression and stability [50, 92–94]. Therefore, we assessed the effect of scPLCL_A2P_ on the total amount of collagen in hUC and hASC monocultures. The amount of collagen in hUC monoculture was lower on scPLCL_A2P_ compared to scPLCL; however, epithelial cells are not expected to produce high quantities of collagen [95, 96]. Instead, the A2P significantly increased the total amount of collagen in hASC monocultures. However, the increase could not be detected in the COL I mRNA levels, yet the amount of hASC COL III mRNA was increased on scPLCL_A2P_. Therefore, as 90% of produced collagen is COL I [50], the effect of A2P in scPLCL_A2P_ seems to be associated with the COL I maturation rather than in the mRNA expression. Previously, supplemented AA or its derivatives have been shown to increase the collagen production in hASCs and bone marrow-derived stem cells [53, 55, 58, 81], and similarly to our findings, COL III mRNA but not COL I mRNA expression was increased in A2P-supplemented osteoblasts [97]. However, Yu et al. reported an increase in both total collagen and COL I mRNA amounts in hASCs cultured with supplemented A2P [53].

Increased amount of COL III may provide more elasticity for the forming tissue [98, 99], but overproduction of COL III may contribute to the formation of fibrosis [99, 100]. Decreased ratio of COL I/III in tissue has been identified in fibrotic growth [101, 102]. Therefore, the amount of COL III formed and the ratio of COL I and COL III in situ are important as abnormal ratio may lead to stiffness or fibrosis in the forming tissue. In our current study, the hASCs COL I/III mRNA ratio was very similar in hASC cultured on scPLCL_A2P_ (0.60) and on scPLCL (0.58). For both, the ratio was less than one, meaning more COL III to COL I mRNA was detected on both scaffolds. The significance of the increased COL III mRNA should be investigated in more detail in future research.

In this study, we established hUC and hASC cocultures to represent the layers of a natural urethra by seeding the cell types on the opposite sides of the scaffold. The hUCs cultured on top of the scaffold represent the urothelium. The scaffold functions as a temporary lamina propria for the hUCs to attach to and the hASCs seeded on the opposite side represent the stromal layer underlying the lamina propria. Previously, hUCs have been cocultured with various cell types, including fibroblasts and smooth muscle cells [103], and hASCs [81, 104]. In our study, we utilized pediatric primary hUCs, whereas in many previously published studies, adult or age unspecified hUCs have been used [85, 86, 104, 105]. However, also the adult-derived hUCs have been demonstrated to retain their capacity to form functional urothelium [86, 105]. Our results show that the hUCs remained viable in the coculture with hASCs on both scPLCL_A2P_ and scPLCL scaffolds. The hUC viability in coculture with hASCs was similar on both scaffolds and appeared alike to the hUCs in monoculture.

Most importantly, our results revealed that the hUCs maintained their phenotype also in the coculture with hASCs. The hUCs on both scaffolds stained positive for pancytokeratin, the cytokeratins presented by epithelial cells. Notably, staining of the late hUC maturation marker UPIII appeared to be higher in the hUCs on the scPLCL_A2P_ and scPLCL scaffolds, whereas only a dim staining was detected on control PS. In a previous study, the expression of UPIII, UPIa, or UPIb was not detected in hUCs under static culture conditions [85] or when cocultured on an ASC-basedself-assembled scaffold [86]. However, when cultured on a combination of fibroblast and ASC-basedself-assembled scaffold, UPIa-, UPIb-, and UPIII-positive hUCs were detected [86]. In contrast, a more recent study reported that hUCs cultured on self-assembled hASC scaffold did maintain their phenotype and expressed the specific markers UPIa, UPIb, CK7, and CK20 [81]. Previously, hUCs have also been cocultured with hASCs to induce hASC urothelial differentiation [104]. The hASCs expressed UPIb and UPII in a direct hUC coculture, whereas they were not detected in an in-direct coculture [104].

We observed that the A2P in scPLCL_A2P_ supported the hASC growth also in coculture despite the used suboptimal coculture medium, whereas on scPLCL, the viable hASCs seem to gradually diminish. Viable hASC sheet is visible on scPLCL_A2P_ also at d14, although the strong hue of hUC staining through the scaffold partly conceals the signal of hASCs. These findings verify the results of the hASC monocultures and are also supported by previous research, where AA and its derivatives have been shown to significantly increase the proliferation rate of human mesenchymal stem cells, including hASCs [53, 58, 106–108]. Additionally, we studied the presence of hASCs in hUC/hASC cocultures with F-actin cytoskeleton staining. This staining also illustrated enhanced hASC growth and spreading on the scPLCL_A2P_ scaffold compared to scPLCL. For the hUCs, actin staining showed similar cortical organization on both scaffolds.

In the current study, more *α*SMA mRNA was measured in hASC monocultures on scPLCL_A2P_, and more *α*SMA-positive hASCs were detected in coculture on scPLCL_A2P_ when compared to scPLCL. Increased *α*SMA expression in ASCs has been linked to enhanced myogenic differentiation [47]. Furthermore, increased hASC and fibroblast *α*SMA expression has been reported when cultured with AA [109, 110]. Therefore, in our study, the A2P in scPLCL_A2P_ may have enhanced the myogenic capacity of hASCs. However, in addition to indicating smooth muscle cell differentiation and presence of smooth muscle cells, *α*SMA is expressed by myofibroblasts [111]. It may also indicate pericytic and proangiogenic functions of hASC [112].

## 5. Conclusions

We demonstrated the potential of scPLCL_A2P_ scaffolds for urethral tissue engineering applications. The novel scPLCL_A2P_ scaffold supported the viability of both hUCs and hASCs in mono- and coculture, even despite the suboptimal coculturing conditions for hASCs. All our results support the cytocompatibility and biocompatibility of the scPLCL_A2P_ scaffolds, yet future in vivo experiments are still required to further ensure their safety. The effect of A2P on hUCs was modest, yet most importantly, the hUCs maintained their phenotype and expressed urothelial maturation markers both in mono- and coculture. We were able to show UPIa, UPIb, and UPIII expression in hUCs; however, further research is needed to explore the hUC maturation in more detail. The proliferation and collagen production of hASCs were significantly increased on scPLCL_A2P_ when compared to scPLCL. However, more research on the effect of A2P on COL I and COL III production is needed to detect and avoid the formation of fibrosis. Moreover, *α*SMA expression in hASC was increased on scPLCL_A2P_, possibly suggesting an increased myogenic or proangiogenic potential. For future research, the coculture conditions need to be optimized to better support both hUCs and hASCs to allow further assessment of hUC maturation and hASC stromal production on the scPLCL_A2P_ scaffolds.

## Figures and Tables

**Table 1:** Expression of cell surface markers on the used hASC donor cell lines after isolation in passage 1 (*P*1).

Cell surface marker expression (%)									
Donor line	*P*	CD14	CD19	CD34	CD45	CD73	CD90	CD105	HLA-DR
hASC 1	1	1.2	0.6	66.7	2	99	99.7	99.7	0.6
hASC 2	1	0.3	0.2	3.7	4.1	81.4	99.2	96.3	0.4
hASC 3	1	1.1	0.8	26.7	1.5	96	98	99.7	1.8

**Table 2:** Primer sequences used in qRT-PCR assay.

Name	Primer	Sequence 5′-3′	Product size (bp)	Accession number
CK7	F	CAT CGA GAT CGC CAC CTA CC	80	NM_005556.3
	R	TAT TCA CGG CTC CCA CTC CA		

CK8	F	CCA TGC CTC CAG CTA CAA AAC	68	M34225.1
	R	AGC TGA GGT TTT ATT TTG GGA CC		

CK19	F	ACT ACA CGA CCA TCC AGG AC	80	NM_002276.4
	R	GTC GAT CTG CAG GAC AAT CC		

UPIa	F	GGG ATC TCC AGT TGT GGT GG	80	NM_007000.3
	R	TCT CAG CAA ACA GGG ACA GG		

UPIb	F	AGT CAC CAA AAC CTG GGA CAG	64	NM_006952.3
	R	TGA TGG ACC ATT TAC GCC ACA		

RPLP0	F	AAT CTC CAG GGG CAC CATT	70	NM_001002
	R	CGC TGG CTC CCA CTT TGT		

*α*SMA	F	GAC AAT GGC TCT GGG CTC TGT AA	194	NM_001613.4
	R	ATG CCA TGT TCT ATC GGG TAC TT		

Elastin	F	GGT GCG GTG GTT CCT CAG CCT GG	613	NM_000501.4
	R	GGG CCT TGA GAT ACC CCA GTG		

COL I	F	CCA GAA GAA CTG GTA CAT CAG CAA	94	NM_000088.3
	R	CGC CAT ACT CGA ACT GGA ATC		

COL III	F	CAG CGG TTC TCC AGG CAA GG	179	NM_000090
	R	CTC CAG TGA TCC CAG CAA TCCC		

**Table 3:** Average scaffold porosity and pore sizes (*n* = 2-3).

	Average porosity (%)	Average pore size	Maximum pore size (*µ*m)
scPLCL_A2P_	63	490 ± 200 *µ*m	840
scPLCL	69	400 ± 140 *µ*m	1060

## Data Availability

The data that support the findings of this study are available from the corresponding author upon reasonable request.
